# Supplementary material for: Modeling glioblastoma heterogeneity as a dynamic network of cell states
Source: Mol Syst Biol. 2021 Sep 16;17(9):e10105. doi: 10.15252/msb.202010105 (PMC8444284; doi:10.15252/msb.202010105)
Supplement: Supplementary file 6 — Source Data for Figure 5 [file MSB-17-e10105-s004.zip › Figure5A_sourcedata/GSEA_3071/nosigdb_stateVI.GseaPreranked.1622795169056/RICHARDS_VERHAAK_CANCERCELL_2010_MESENCHYMAL.html]

Details for gene set RICHARDS\_VERHAAK\_CANCERCELL\_2010\_MESENCHYMAL[GSEA]

|  || Dataset | state63071 |
| Phenotype | NoPhenotypeAvailable |
| Upregulated in class | na\_pos |
| GeneSet | RICHARDS\_VERHAAK\_CANCERCELL\_2010\_MESENCHYMAL |
| Enrichment Score (ES) | 0.41627425 |
| Normalized Enrichment Score (NES) | 2.8338125 |
| Nominal p-value | 0.0 |
| FDR q-value | 0.0 |
| FWER p-Value | 0.0 |
Table: GSEA Results Summary

  

Fig 1: Enrichment plot: RICHARDS\_VERHAAK\_CANCERCELL\_2010\_MESENCHYMAL      
 Profile of the Running ES Score & Positions of GeneSet Members on the Rank Ordered List

  

| PROBE | GENE SYMBOL | GENE\_TITLE | RANK IN GENE LIST | RANK METRIC SCORE | RUNNING ES | CORE ENRICHMENT || 1 | SERPINE1 |  |  | 0 | 1.673 | 0.0423 | Yes |
| 2 | THBS1 |  |  | 12 | 1.097 | 0.0670 | Yes |
| 3 | DCBLD2 |  |  | 16 | 1.066 | 0.0931 | Yes |
| 4 | LOX |  |  | 29 | 0.968 | 0.1142 | Yes |
| 5 | PLAUR |  |  | 34 | 0.936 | 0.1368 | Yes |
| 6 | ARHGAP29 |  |  | 54 | 0.863 | 0.1533 | Yes |
| 7 | DAB2 |  |  | 78 | 0.788 | 0.1668 | Yes |
| 8 | ANXA2 |  |  | 79 | 0.783 | 0.1866 | Yes |
| 9 | LTBP1 |  |  | 84 | 0.779 | 0.2052 | Yes |
| 10 | UAP1 |  |  | 93 | 0.742 | 0.2217 | Yes |
| 11 | FXYD5 |  |  | 107 | 0.732 | 0.2366 | Yes |
| 12 | CLEC2B |  |  | 132 | 0.688 | 0.2472 | Yes |
| 13 | MYOF |  |  | 157 | 0.655 | 0.2571 | Yes |
| 14 | PYGL |  |  | 177 | 0.622 | 0.2675 | Yes |
| 15 | MAN1A1 |  |  | 250 | 0.563 | 0.2615 | Yes |
| 16 | CLIC1 |  |  | 258 | 0.561 | 0.2737 | Yes |
| 17 | LRRFIP1 |  |  | 268 | 0.555 | 0.2852 | Yes |
| 18 | SP100 |  |  | 294 | 0.544 | 0.2920 | Yes |
| 19 | MYH9 |  |  | 312 | 0.534 | 0.3007 | Yes |
| 20 | PROCR |  |  | 314 | 0.532 | 0.3139 | Yes |
| 21 | LGALS1 |  |  | 323 | 0.526 | 0.3250 | Yes |
| 22 | IGFBP6 |  |  | 341 | 0.515 | 0.3333 | Yes |
| 23 | CNN2 |  |  | 349 | 0.512 | 0.3442 | Yes |
| 24 | RAB32 |  |  | 352 | 0.512 | 0.3566 | Yes |
| 25 | FHL2 |  |  | 397 | 0.491 | 0.3567 | Yes |
| 26 | RELB |  |  | 406 | 0.486 | 0.3668 | Yes |
| 27 | ASL |  |  | 421 | 0.482 | 0.3750 | Yes |
| 28 | TNFAIP3 |  |  | 445 | 0.473 | 0.3806 | Yes |
| 29 | COPZ2 |  |  | 457 | 0.469 | 0.3893 | Yes |
| 30 | CTSB |  |  | 471 | 0.464 | 0.3974 | Yes |
| 31 | BNC2 |  |  | 477 | 0.462 | 0.4077 | Yes |
| 32 | RBKS |  |  | 501 | 0.454 | 0.4127 | Yes |
| 33 | RRAS |  |  | 555 | 0.438 | 0.4090 | Yes |
| 34 | SLC16A3 |  |  | 569 | 0.433 | 0.4163 | Yes |
| 35 | MVP |  |  | 642 | 0.413 | 0.4065 | No |
| 36 | RBMS1 |  |  | 678 | 0.405 | 0.4070 | No |
| 37 | RAB11FIP1 |  |  | 706 | 0.395 | 0.4094 | No |
| 38 | ARPC1B |  |  | 847 | 0.369 | 0.3795 | No |
| 39 | SHC1 |  |  | 913 | 0.358 | 0.3703 | No |
| 40 | EHD2 |  |  | 1030 | 0.340 | 0.3464 | No |
| 41 | CAST |  |  | 1070 | 0.334 | 0.3439 | No |
| 42 | ICAM3 |  |  | 1177 | 0.319 | 0.3223 | No |
| 43 | EMP3 |  |  | 1278 | 0.306 | 0.3020 | No |
| 44 | RHOG |  |  | 1355 | 0.297 | 0.2882 | No |
| 45 | CEBPB |  |  | 1389 | 0.293 | 0.2864 | No |
| 46 | DSE |  |  | 1484 | 0.282 | 0.2671 | No |
| 47 | MAN2A1 |  |  | 1507 | 0.279 | 0.2680 | No |
| 48 | HEXB |  |  | 1568 | 0.272 | 0.2581 | No |
| 49 | POLD4 |  |  | 1579 | 0.271 | 0.2621 | No |
| 50 | CLCF1 |  |  | 1592 | 0.269 | 0.2656 | No |
| 51 | ACSL1 |  |  | 1605 | 0.268 | 0.2690 | No |
| 52 | MGAT1 |  |  | 1649 | 0.264 | 0.2636 | No |
| 53 | FURIN |  |  | 1702 | 0.260 | 0.2556 | No |
| 54 | SLC10A3 |  |  | 1708 | 0.260 | 0.2608 | No |
| 55 | PLK3 |  |  | 1710 | 0.259 | 0.2671 | No |
| 56 | CTSC |  |  | 1767 | 0.254 | 0.2578 | No |
| 57 | YAP1 |  |  | 1783 | 0.252 | 0.2600 | No |
| 58 | SAT1 |  |  | 1952 | -0.271 | 0.2198 | No |
| 59 | TGOLN2 |  |  | 2055 | -0.288 | 0.1984 | No |
| 60 | ZNF217 |  |  | 2192 | -0.313 | 0.1682 | No |
| 61 | SFT2D2 |  |  | 2235 | -0.320 | 0.1646 | No |
| 62 | FNDC3B |  |  | 2645 | -0.410 | 0.0603 | No |
| 63 | TGFBI |  |  | 2679 | -0.419 | 0.0617 | No |
| 64 | EFEMP2 |  |  | 3010 | -0.546 | -0.0170 | No |
| 65 | GRN |  |  | 3016 | -0.547 | -0.0046 | No |
| 66 | ITGA4 |  |  | 3132 | -0.610 | -0.0214 | No |
| 67 | HEXA |  |  | 3370 | -0.840 | -0.0666 | No |
| 68 | MRC2 |  |  | 3439 | -0.981 | -0.0608 | No |
| 69 | WWTR1 |  |  | 3519 | -1.185 | -0.0530 | No |
| 70 | PDPN |  |  | 3580 | -1.590 | -0.0296 | No |
| 71 | COL1A2 |  |  | 3602 | -1.803 | 0.0101 | No |
Table: GSEA details [plain text format]

  

Fig 2: RICHARDS\_VERHAAK\_CANCERCELL\_2010\_MESENCHYMAL: Random ES distribution      
 Gene set null distribution of ES for **RICHARDS\_VERHAAK\_CANCERCELL\_2010\_MESENCHYMAL**
